# The lipophilic cyclic peptide cyclosporin A induces aggregation of gel-forming mucins

**DOI:** 10.1038/s41598-022-10125-y

**Published:** 2022-04-13

**Authors:** Hisanao Kishimoto, Caroline Ridley, David J. Thornton

**Affiliations:** 1 Department of Biopharmaceutics, School of Pharmacy, Tokyo University of Pharmacy and Life Sciences, 1432-1, Horinouchi, Hachioji, Tokyo 192-0392 Japan; 2 Wellcome Trust Centre for Cell-Matrix Research, School of Biological Sciences, Faculty of Biology, Medicine and Health, Manchester Academic Health Sciences Centre, The University of Manchester, Oxford Road, Manchester, M13 9PT UK

**Keywords:** Glycobiology, Glycobiology

## Abstract

Cyclic peptides are good candidates for orally delivered therapeutics, however, issues remain in their development due to low intestinal permeability. Although some of the biological factors have been reported that regulate intestinal permeation of cyclic peptides, the influence of the mucus barrier, a major hurdle to epithelial drug delivery, on cyclic peptide bioavailability is unclear. In this study, we show that the lipophilic cyclic peptide, cyclosporin A (CsA), interacted with, and likely induced aggregation, of polymeric, gel-forming mucins (MUC2, MUC5AC and MUC5B) which underpin the mucus gel-networks in the gastrointestinal tract. Under similar conditions, two other cyclic peptides (daptomycin and polymyxin B) did not cause mucin aggregation. Using rate-zonal centrifugation, purified MUC2, MUC5AC and MUC5B mucins sedimented faster in the presence of CsA, with a significant increase in mucins in the pellet fraction. In contrast, mucin sedimentation profiles were largely unaltered after treatment with daptomycin or polymyxin B. CsA increased MUC5B sedimentation was concentration-dependent, and sedimentation studies using recombinant mucin protein domains suggests CsA most likely causes aggregation of the relatively non-O-glycosylated N-terminal and C-terminal regions of MUC5B. Furthermore, the aggregation of the N-terminal region, but not the C-terminal region, was affected by pH. CsA has partially N-methylated amide groups, this unique molecular structure, not present in daptomycin and polymyxin B, may potentially be involved in interaction with gel-forming mucin. Taken together, our results indicate that the interaction of gel-forming mucins with the cyclic peptide CsA is mediated at the N- and C-terminal domains of mucin polymers under physiological conditions. Our findings demonstrate that the mucus barrier is an important physiological factor regulating the intestinal permeation of cyclic peptides in vivo.

## Introduction

Therapeutic macromolecular compounds, such as proteins, peptides, nucleic acids, and oligosaccharides are increasingly anticipated as novel drugs for a wide range of diseases^1^. Drug discovery has traditionally focused on synthesis of hydrophilic and highly absorbable small molecules (~ 0.5 kDa) as oral drug therapeutics, but relatively low molecular weight peptide and protein-like compounds (i.e., within the range of 0.5–5.0 kDa) are attractive alternatives due to their specificity, efficacy, and low toxicity compared to synthetic small molecule drugs. Moreover, low molecular weight peptide and protein-like compounds may also offer other advantages for example, the relatively low cost to produce compared to high-molecular-weight drugs (~ 150 kDa) such as large-scale protein/antibody production. In particular, cyclic peptides combine several properties such as high affinity, target selectivity and stability (enzymatic and chemical) which are important properties for therapeutics and make them ideal as orally delivered candidate drugs^1,2^. Most clinically approved cyclic peptides are currently derived from natural products, thus it is likely that novel cyclic peptides will be a major drug development target in future. However, several important issues remain in the development of orally delivered cyclic peptide therapeutics, the most important one is poor bioavailability due to low intestinal permeability^3,4^. Therefore, it is important to clarify the physiological factors that regulate the intestinal permeation of cyclic peptides.

Cyclic peptides are mainly absorbed via passive diffusion across the intestinal epithelial membrane after oral administration, which is a similar process to most lipophilic small-molecule drugs^5^. However, it is important to consider various absorption mechanisms because different factors are likely to have differing effects on cyclic peptide permeability. For instance, assuming sufficient dissolution of cyclic peptides in the intestinal lumen, most of these peptides are likely to interact with various components of gastrointestinal fluids before reaching the epithelial cell surface. Although several biological factors have been reported that regulate the intestinal permeation process of cyclic peptides, the influence of the mucus barrier on the absorption of cyclic peptides still remains unclear.

Mucus covers the surface of secretory epithelia such as intestinal and respiratory epithelium, and provides a dynamic barrier to protect against infections and foreign particles under physiological conditions^6–8^. This complex hydrogel is composed of many components including water, ions and hundreds of proteins, but the major structural components are the mucins, which are polymeric, high-molecular weight, extensively O-glycosylated proteins^6,7^. Mucins form gel-like networks with viscoelastic properties that act as a biological molecular sieve with a range of pore sizes between 20 and 400 nm, allowing the exclusion of foreign particles such as toxins, pathogens and nanomaterials^8^. Moreover, due to their complex chemical composition, mucins can also act as a selective physicochemical barrier via binding to their negatively charged O-glycans clustered in the heavily glycosylated central domains and to hydrophobic, non-O-glycosylated cysteine-rich domains^6,7^. These barrier properties of mucins are key factors to be considered in the intestinal absorption of drugs^9^. However, mucus is a dynamic barrier, and its composition (in particular, mucin composition) and biological functions differ depending on the mucosal tissue^7,8^. Although, mucins potentially act as important physiological regulators of intestinal drug absorption (including cyclic peptides) the molecular basis of this regulation is incompletely defined. Therefore, it is important to characterize the effect of different mucins on cyclic peptide diffusion to elucidate their potential influence on bioavailability and drug absorption.

Mucins are a large family of glycoproteins which is divided into two types: the secreted mucins (gel-forming mucins: MUC2, 5AC, 5B, 6, and 19, non-gel-forming mucins: MUC7) and the membrane-bound mucins (transmembrane mucins: MUC1, 3A/B, 4, 12, 13, 15, 16, 17, and 20)^10,11^. The membrane-bound mucins form part of the glycocalyx layer on the surface of epithelial cells; whereas, the polymeric gel-forming mucins, which are the major focus of this study, underpin mucus gel-networks and can potentially limit the diffusion of drugs toward the epithelium^12^. Recent pharmaceutical studies have demonstrated that mucus-penetrative and mucoadhesive drug carrier technologies can improve the delivery of orally delivered cyclic peptides through mucus^13,14^. However, there are no reports that have described the interaction between the polymeric mucins which underpin mucus barriers and cyclic peptides during intestinal absorption process at the molecular level.

Therefore, in this study, we have investigated the interaction between several cyclic peptides and three different polymeric mucins that are key components of saliva (MUC5B), intestinal (MUC2) and gastric mucus (MUC5AC). Here, we selected cyclic peptides (daptomycin, polymyxin B and cyclosporin A) known to be derived from natural products with different physicochemical properties (e.g., lipophilicity and physiological charge), and investigated their interaction with purified human polymeric mucins (MUC2, MUC5AC and MUC5B). We examined the sedimentation behavior of polymeric mucins to evaluate structural changes in the mucin network engendered by interaction with cyclic peptides. Moreover, the location of the cyclic peptide-interaction sites on polymeric mucin was explored using the recombinant N-terminal, C-terminal and CysD protein sub-domains of MUC5B. We found that interaction with cyclosporin A, but not daptomycin and polymyxin B, resulted in aggregation of MUC2, MUC5AC and MUC5B, which may impact mucin viscoelastic properties and the bioavailability of cyclosporin A after oral administration.

## Results

### The effect of cyclic peptides on the sedimentation behavior of purified polymeric mucins

Prior to sedimentation analyses, the mucins present in the purified samples from the 3 cell lines was assessed by using MUC2, MUC5AC and MUC5B specific antibodies and PAS staining (Figs. S1 and S2). This confirmed that MUC2, MUC5AC and MUC5B were the predominant mucins in the preparations purified from LS174T, MUC5B knock-down A549 and MUC5AC knock-down A549 cell lines, respectively. It is important to note that while it has been reported that MUC2 is the predominant mucin expressed by LS174T cells, this cell line also expresses MUC6, but at a lower level^15^. However, due to lack of a MUC6 specific probe, we were unable to assess the MUC6 content of the LS174T mucin preparation.

**Figure 1:**
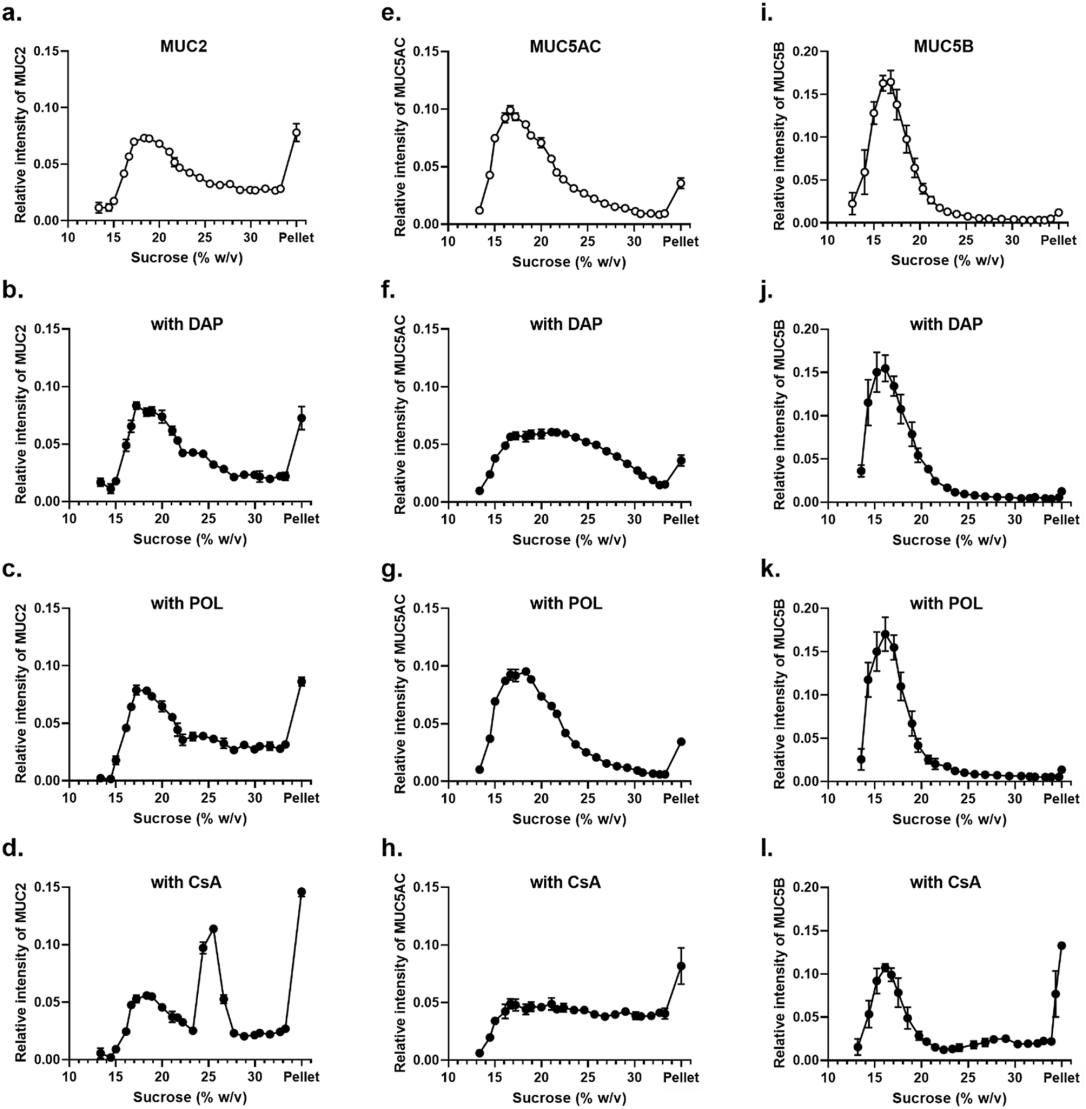
Sedimentation profiles of purified gel-forming mucins in the presence of cyclic peptide. Rate-zonal centrifugation in 10–35% (w/v) sucrose gradients of purified MUC2 (**a**–**d**), MUC5AC (**e**–**h**), and MUC5B (i–**l**), untreated (**a**, **e**, **i**), with 1 mM DAP (**b**, **f**, **j**), with POL (**c**, **g**, **k**), or with CsA (**d**, **h**, **l**). Mucins were detected in sucrose gradient fractions after slot blotting by PAS staining (MUC2; **a**–**d**) or the mucin-specific antibody probes, MAN-5ACI (**e**–**h**), and EUMUC5B (**i**–**l**). Band intensities were quantified using the Odyssey Imaging system. The results are presented as the mean ± s.e.m. (n = 3–5) from 3 independent experiments.

To determine whether cyclic peptides could influence the biophysical properties of polymeric mucins, we assessed changes in the sedimentation behavior of purified MUC2, MUC5AC and MUC5B mucins after incubation with either 1 mM daptomycin (DAP), 1 mM polymyxin B sulphate (POL), or 1 mM cyclosporin A (CsA) by using rate-zonal centrifugation on 10–35% (w/v) sucrose gradients (Fig. 1). In the absence of cyclic peptide, polymeric mucins were found mainly in the fractions containing 10–25% (w/v) sucrose, and only a small amount was pelleted (Fig. 1a,e,i). After incubation with 1 mM CsA, the mucin sedimentation was altered with a higher proportion of the mucins in the fractions containing 25–35% (w/v) sucrose, as well as a significant increase of mucins in the pellet (Fig. 1d, h, l). The percentage of MUC2, MUC5AC, and MUC5B in the pellet were 14.6% increased from 7.8% without CsA (Table S1), 8.2% increased from 3.6% without CsA (Table S2) and 13.3% increased from 1.2% without CsA (Table 1), respectively. These results suggested that interaction with CsA induced mucin aggregate formation which increased sedimentation rate. In marked contrast, the polymeric mucin sedimentation profiles were largely unaltered after incubation with 1 mM DAP and 1 mM POL (Fig. 1b,c,g,j,k; Tables S1, S2 and S3). However, incubation of MUC5AC with 1 mM DAP resulted in a broader MUC5AC sedimentation profile (Fig. 1f; Table S2).

**Table 1:** Percentage of MUC5B in sucrose gradient fractions (CsA concentration-dependence).

Sucrose (% w/v)	Cyclosporin A			
	Control	200 μM	1 mM	2 mM
10–15	10.24 ± 3.51	9.54 ± 2.35	9.01 ± 1.31	12.47 ± 2.32
15–20	74.07 ± 5.58	63.68 ± 4.02	43.85 ± 0.75**	35.25 ± 0.75**
20–25	10.41 ± 3.27	10.43 ± 1.80	7.73 ± 1.56	12.03 ± 2.53
25–30	2.25 ± 0.82	5.72 ± 1.46	8.54 ± 2.27*	14.22 ± 1.63**
30–35	1.83 ± 1.03	5.04 ± 1.24	16.40 ± 3.18**	14.27 ± 2.68**
Pellet	1.20 ± 0.25	5.59 ± 3.20	13.27 ± 0.32**	11.77 ± 1.33**

Results are presented as the mean ± s.e.m. (n = 3–5) from 3 independent experiments.

**Figure 2:**
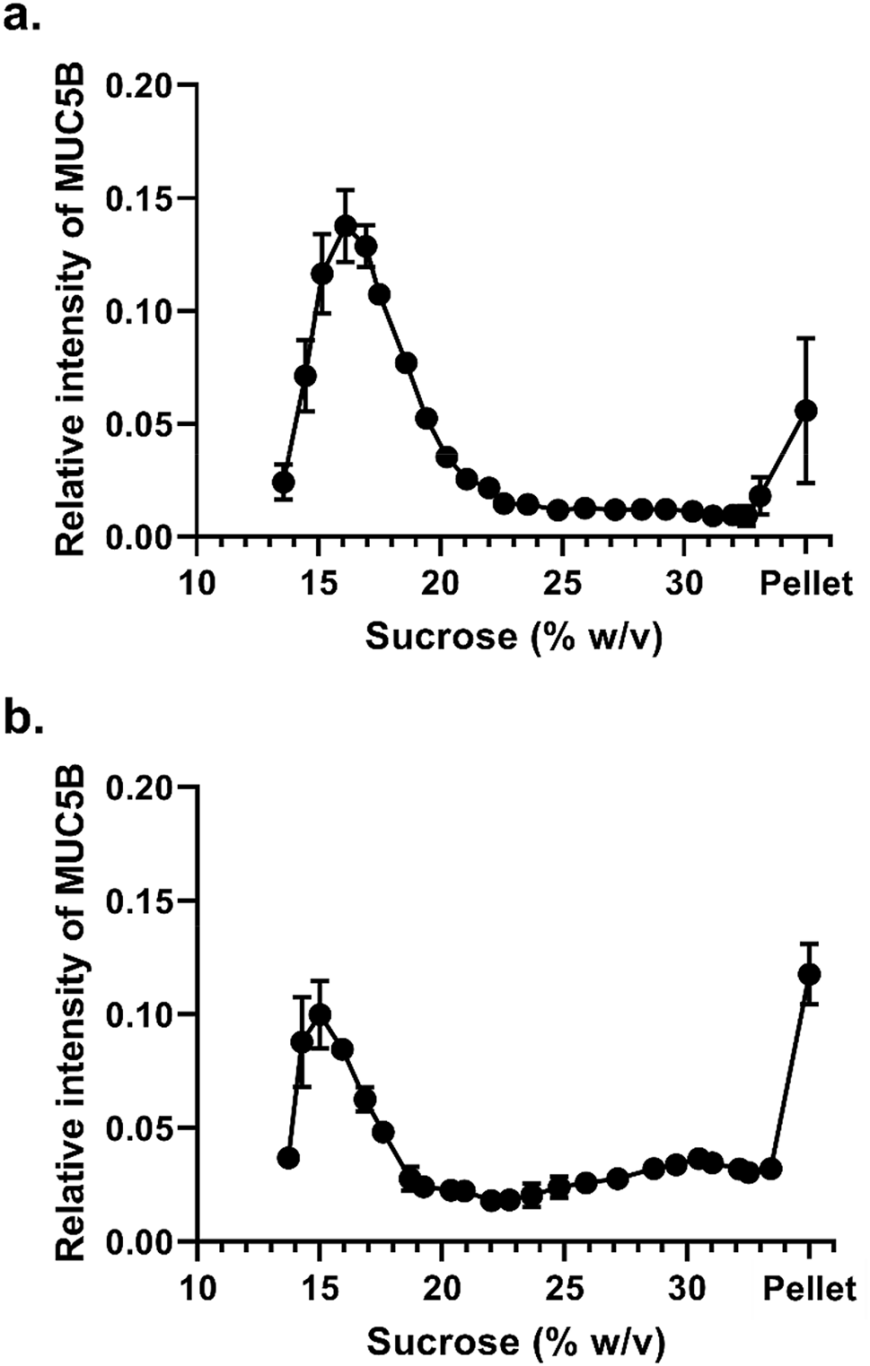
Dose-dependence of CsA on MUC5B sedimentation behavior. Rate-zonal centrifugation in 10–35% (w/v) sucrose gradients of purified MUC5B (200 μg/mL) with 200 μM CsA (**a**) and 2 mM CsA (**b**). Mucins were detected in sucrose gradient fractions after slot blotting by the mucin-specific antibody probe, EUMUC5B. Band intensities were quantified using the Odyssey Imaging system. The results are presented as the mean ± s.e.m. (n = 3–4) from 3 independent experiments.

*P < 0.05, **P < 0.01 compared with control condition (ANOVA followed by Dunnett’s method). The percentage of MUC5B in fractions across the sucrose gradient was calculated from Figs. 1 and 2.

To further evaluate the effect of the cyclic peptide CsA on mucin properties, we examined how CsA influenced the sedimentation behavior of purified MUC5B polymers and recombinant MUC5B protein sub-domains.

### Concentration-dependence of the interaction between MUC5B and CsA

**Figure 3:**
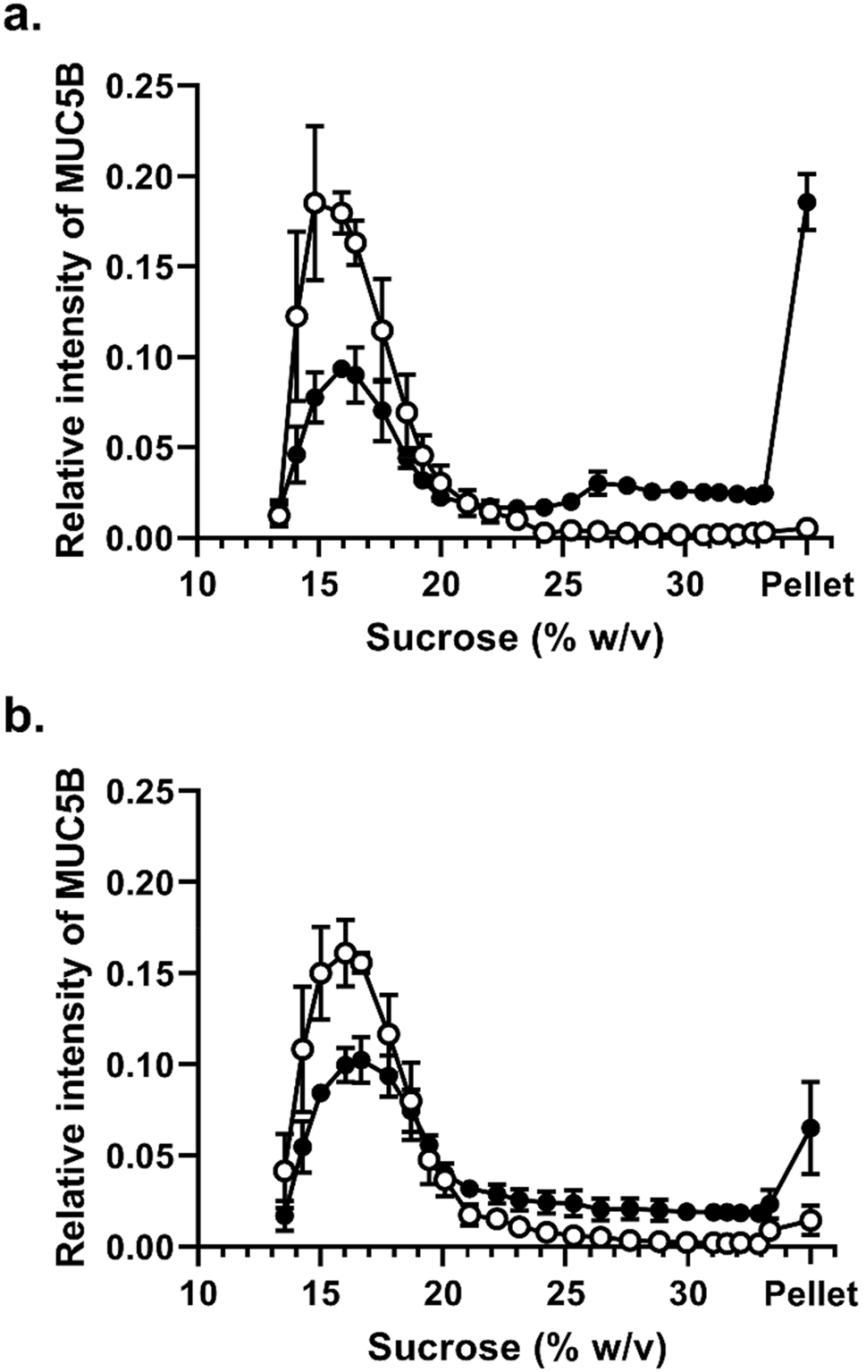
Concentration-dependence of MUC5B on CsA mediated aggregation. Rate-zonal centrifugation in 10–35% (w/v) sucrose gradients of purified MUC5B (**a** 100 μg/mL, **b** 400 μg/mL) alone (white) or with 1 mM CsA (black). Mucins were detected in sucrose gradient fractions after slot blotting by the mucin-specific antibody probe, EUMUC5B. Band intensities were quantified using the Odyssey Imaging system. The results are presented as the mean ± s.e.m. (n = 3) from 3 independent experiments.

To investigate the concentration dependence of CsA on MUC5B polymer aggregation, we used two further concentrations of CsA (200 μM and 2 mM) at a fixed MUC5B concentration (200 μg/mL) (Fig. 2 and Table 1). The proportion of MUC5B in the fractions at the bottom half of the sucrose gradients, including the pellet, was increased at the higher concentration of CsA, indicating a concentration-dependent effect. The increased sedimentation rate of MUC5B in the presence of CsA suggests aggregation of MUC5B. We next examined the effect on MUC5B sedimentation profile at a constant concentration of CsA (1 mM) while varying MUC5B concentration (100 and 400 μg/mL) (Fig. 3 and Table 2). The data showed the proportion of MUC5B in the fractions at the bottom half of the gradient (and pellet) was decreased at the higher concentration of MUC5B, clearly demonstrating the concentration-dependence of the effect and suggesting the aggregation is dependent on the ratio of the CsA to mucin concentration. The alteration of MUC5B sedimentation profiles by CsA suggests that the lipophilic cyclic peptide can change MUC5B network properties.

**Table 2:** Percentage of MUC5B in sucrose gradient fractions (MUC5B concentration dependence).

Sucrose (% w/v)	MUC5B 100 μg/mL		MUC5B 400 μg/mL	
	Control	CsA 1 mM	Control	CsA 1 mM
10–15	16.84 ± 1.76	7.82 ± 0.55**	14.99 ± 5.45	7.18 ± 2.22
15–20	75.44 ± 0.63	40.63 ± 1.60**	69.67 ± 2.45	55.01 ± 4.59*
20–25	4.66 ± 1.62	8.04 ± 0.48	6.93 ± 2.93	11.41 ± 1.70
25–30	1.46 ± 0.20	12.68 ± 0.28**	1.71 ± 0.40	10.09 ± 3.10
30–35	1.13 ± 0.35	12.26 ± 0.78**	1.66 ± 0.58	9.79 ± 1.11**
Pellet	0.55 ± 0.10	18.56 ± 1.55**	1.44 ± 0.81	6.52 ± 2.52

Results are presented as the mean ± s.e.m. (n = 3) from 3 independent experiments.

*P < 0.05, **P < 0.01 compared with control condition (ANOVA followed by Dunnett’s method). The percentage of MUC5B in fractions across the sucrose gradient was calculated from Fig. 3.

While these results showed that CsA has ability to alter the polymeric MUC5B network, we have not demonstrated which domain(s) of the MUC5B molecule is responsible. Therefore, to investigate this further, we expressed recombinant N-terminal (NT5B), C-terminal (CT5B) and CysD (Cys7) sub-domains of MUC5B and evaluated whether CsA influenced the sedimentation profile of these protein domains.

### The effect of CsA on the sedimentation behavior of MUC5B protein sub-domains

**Figure 4:**
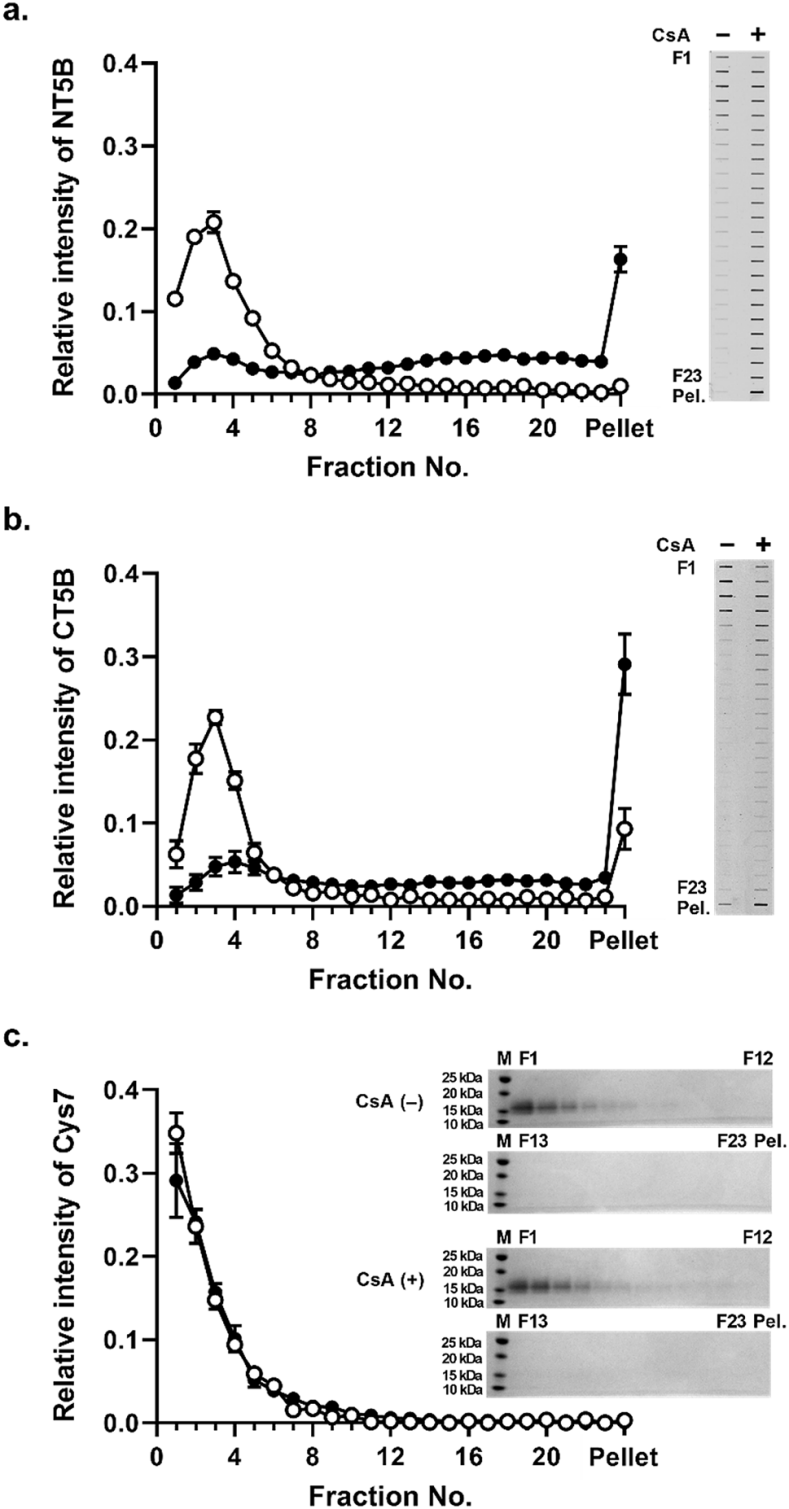
Sedimentation behavior of MUC5B protein sub-domains in the presence of CsA. Rate-zonal centrifugation in 5–20% (w/v) sucrose gradients of NT5B (**a**), CT5B (**b**), and Cys7 (**c**) recombinant proteins alone (white) or with 1 mM CsA (black). In (**a**,**b**), NT5B and CT5B proteins were immunodetected in the gradient fractions after slot blotting using anti-6X His-tag antibody. In (**c**), Cys 7 protein was detected by SDS-PAGE and band intensity measured after InstantBlue staining. The results are presented as the mean ± s.e.m. (n = 3–6) from 3 independent experiments. *Pel.* pelleted sample. The blots were cropped and full-length blots are presented in Supplementary Fig. S3.

The sedimentation behavior of the expressed recombinant N-terminal protein of MUC5B (NT5B) was analyzed in the absence and presence of CsA, by rate-zonal centrifugation on 5–20% (w/v) sucrose gradients. Untreated NT5B was mainly present in fractions 1–5 (Fig. 4a) which contained greater than 70% of the NT5B protein (Table 3). In contrast, the presence of 1 mM CsA, resulted in a marked change in the sedimentation profile (Fig. 4a). Although the treated NT5B was not completely aggregated, the NT5B protein exhibited a broader sedimentation profile, and the amount of NT5B in the pellet was increased tenfold compared to the control, untreated NT5B (Table 3).

**Table 3:** Percentage of NT5B and CT5B in sucrose gradient fractions.

Fraction number	NT5B 400 μg/mL		CT5B 400 μg/mL	
	Control	CsA 1 mM	Control	CsA 1 mM
1–5	74.19 ± 1.55	17.50 ± 0.32**	68.22 ± 2.24	18.91 ± 5.17**
6–10	14.16 ± 1.39	13.01 ± 1.10	10.47 ± 1.59	14.57 ± 1.68
11–15	5.82 ± 0.20	18.38 ± 1.23**	4.93 ± 0.34	13.38 ± 1.19
16–20	3.76 ± 0.82	22.43 ± 1.29**	4.25 ± 1.16	15.27 ± 3.04**
21–23	1.08 ± 0.22	12.38 ± 0.69**	2.84 ± 0.67	8.79 ± 1.97**
Pellet	1.00 ± 0.47	16.31 ± 1.52**	9.30 ± 2.45	29.09 ± 3.65**

Results are presented as the mean ± s.e.m. (n = 3–6) from 3 independent experiments.

*P < 0.05, **p < 0.01 compared with control condition (ANOVA followed by Dunnett’s method). The percentage of NT5B and CT5B in fractions across the sucrose gradient was calculated from Fig. 4.

Similar results were obtained using the recombinant C-terminal protein of MUC5B (CT5B) (Fig. 4b and Table 3). These results suggested that the effect of CsA on CT5B may be through a similar aggregation mechanism as observed for NT5B. Subsequently, we examined the effect of CsA on the Cys7 recombinant protein, one of the seven CysD domains of MUC5B, which are highly homologous and highly conserved between polymeric mucins, and may act as points for mucin cross-linking^16^. Rate-zonal centrifugation of recombinant Cys7 protein, both in the absence and presence of 1 mM CsA, showed no marked difference in sedimentation profile of the Cys7 protein (Fig. 4c) and revealed that more than 80% of Cys7 was mainly contained in fractions 1–5 under both conditions (Table 3). While it cannot be ruled out that there is an interaction between Cys7 protein of MUC5B and CsA, our data showed that such an interaction does not lead to a change in sedimentation profile of the Cys7 protein (at least under the conditions of the experiment).

### The effect of pH on the interactions between MUC5B and CsA

**Figure 5:**
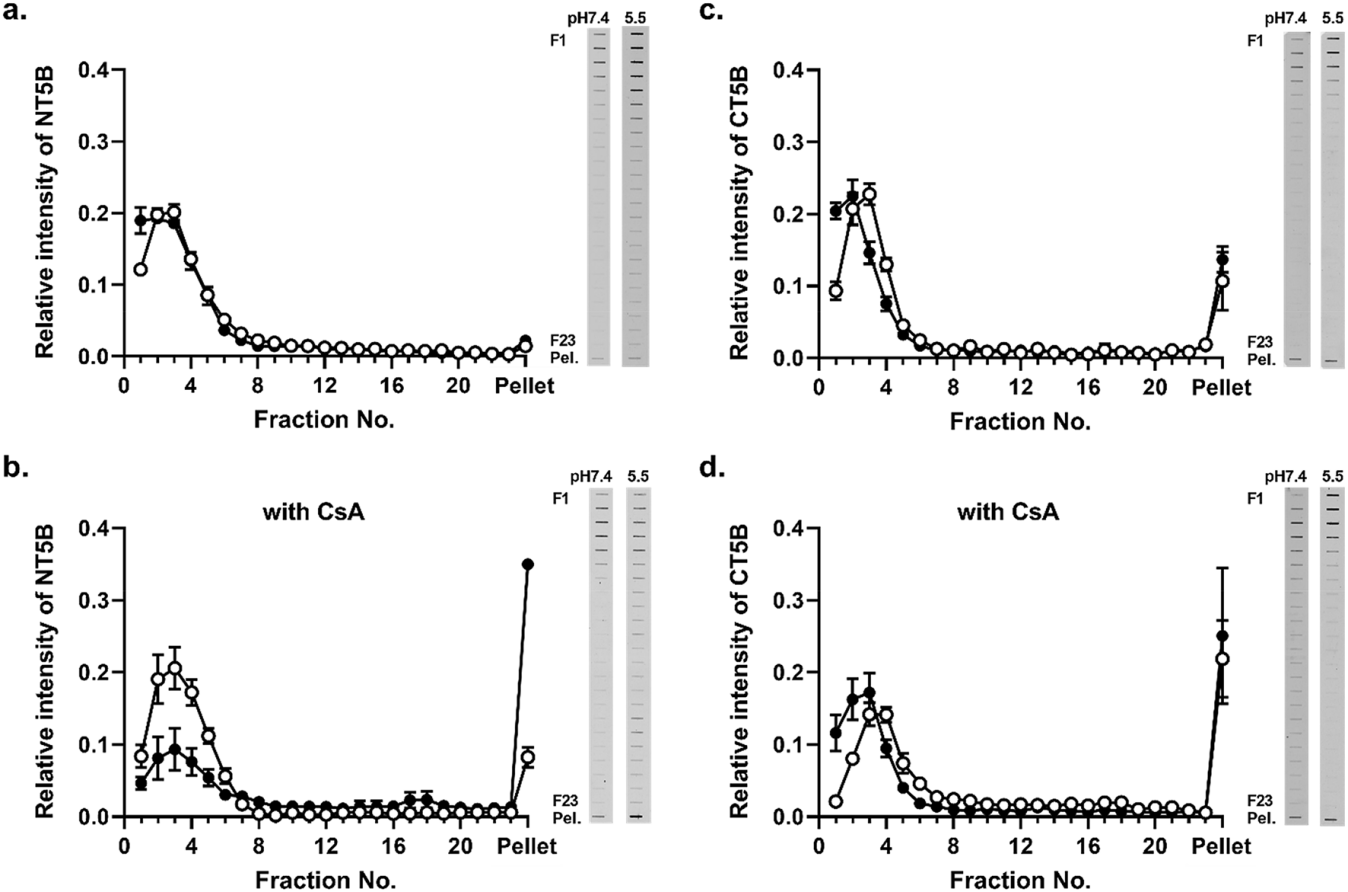
The effect of pH on the aggregation of MUC5B sub-domains in the presence of CsA. Rate-zonal centrifugation on 5–20% (w/v) sucrose gradients of NT5B (**a**,**b**) and CT5B (**c**,**d**) recombinant protein at pH 7.4 (white) and pH 5.5 (black) in PBS (**a**,**c**) or in PBS containing 200 μM CsA (**b**,**d**). Proteins were immunodetected in the gradient fractions using an anti-6X His-tag antibody. The results are presented as the mean ± s.e.m. (n = 3–5) from 3 independent experiments. The blots were cropped and full-length blots are presented in Supplementary Fig. S4.

**Figure 6:**
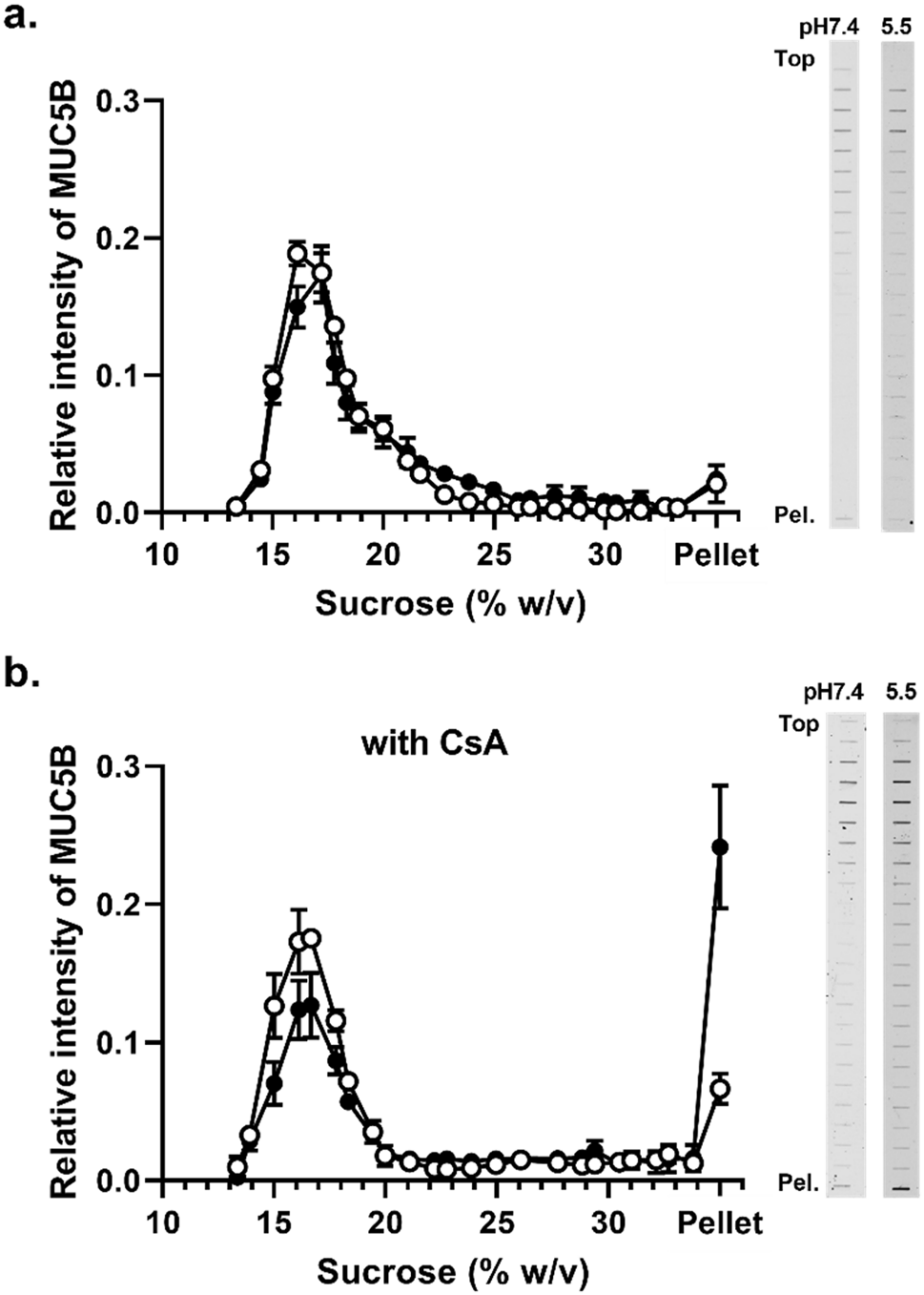
The effect of pH on the aggregation of purified MUC5B in the presence of CsA. Rate-zonal centrifugation on 10–35% (w/v) sucrose gradients of purified MUC5B at pH 7.4 (white) and pH 5.5 (black) in PBS (**a**) or in PBS containing 200 μM CsA (**b**). MUC5B was immunodetected in the gradient fractions using the EUMUC5B antibody after slot blotting. The results are presented as the mean ± s.e.m. (n = 3–6) from 3 independent experiments. The blots were cropped and full-length blots are presented in Supplementary Fig. S5.

The physiological pH values in gastrointestinal tract vary from acidic (~ pH 1–2 in the stomach) to neutral (pH 6.5–7.4 for the mouth, and small and large intestine), however the pH value of the mucus layer at the surface of intestinal epithelium is lower than that of the gastrointestinal fluid at pH 5.5^17,18^. Therefore, we investigated the effect of pH on the CsA-dependent increase sedimentation rate of the recombinant MUC5B protein domains (Fig. 5) and native MUC5B polymers (Fig. 6). These experiments were performed using 200 μM CsA at pH 7.4 and 5.5.

Lowering the pH resulted in no change in NT5B protein sedimentation profile in the absence of CsA, thus pH alone did not influence NT5B self-association (Fig. 5a). Recombinant NT5B protein in the presence of CsA at pH 5.5, showed increased sedimentation compared to the corresponding treatment at pH 7.4, with ~ 4.2-fold higher NT5B protein found in the pellet (Fig. 5b and Table S4). These results showed the interaction between NT5B and CsA was pH-dependent and suggested that decreasing the pH to 5.5 increased the affinity of the interaction. In contrast, there was no change in the CT5B protein sedimentation profile after lowering the pH from 7.4 to 5.5 (Fig. 5c,d), which suggested that this change in pH did not increase aggregation.

Finally, we evaluated the effect of pH on the CsA-dependent aggregation of native, polymeric MUC5B (Fig. 6). In the absence of CsA, the sedimentation profile of MUC5B was not altered at either pH 7.4 or pH5.5 (Fig. 6a). Lowering the pH in the presence of 200 μM CsA resulted in a significant change in the sedimentation profile of MUCB (Fig. 6b). Furthermore, there was a ~ 3.6-fold increase in MUC5B in the pellet at pH 5.5 compared to pH 7.4 (Table S5). These data indicate that the interaction between CsA and polymeric mucins is likely to occur in the mucus layer at the surface of the intestinal epithelium under physiological conditions.

### Fluorescence spectral analysis of the interaction between MUC5B and CsA

Our results have identified that MUC5B interacts with CsA to alter the mucin sedimentation profile and that the N- and C-terminal protein domains of MUC5B were important sites of interaction. We confirmed the interaction by using fluorescence spectral analysis of the purified MUC5B polymer in the presence of a range of concentrations of CsA (0–200 μM; Fig. S6). We found that the fluorescence intensity of MUC5B was reduced with increasing concentrations of CsA showing binding of CsA to the protein-domains of the MUC5B polymer.

## Discussion

The polymeric mucins underpin the mucus gel network and likely limit drug diffusion toward the surface of intestinal epithelial cells, although the molecular basis of this regulation has remained unclear^5,13^. In this study, we have demonstrated that CsA interacted with, and likely induced aggregation of polymeric mucins (MUC2, MUC5AC and MUC5B). Furthermore, we revealed that CsA likely induces aggregation by interacting mainly with the N-terminal and C-terminal protein domains of mucins. Similar observations have been reported for green tea polyphenols and polymeric mucins^19,20^. Therefore, it seems reasonable to conclude that mucins will influence the bioavailability of lipophilic cyclic peptide drugs such as CsA and their ability to diffuse through the mucus barrier to access the underlying epithelial cells. Our results thus mark a major extension of our understanding of the way in which mucins have controlling effects on mucosal layer barrier function.

Although, the detailed molecular mechanism for the influence of polymeric mucins on the intestinal drug absorption process has still to be clarified, our results provide new insight into this process. For drug compounds to be absorbed across intestinal epithelia, they have to permeate through the mucin-rich mucus layer that covers the surface of the gastrointestinal epithelium which acts as a dynamic barrier to protect against xenobiotics^7,12^. Mucins form entangled and cross-linked networks that contribute to the selective physicochemical barrier properties of mucus by acting as molecular sieves and/or providing binding sites^8^. Thus, diffusion of a drug through mucin networks depends on its molecular size and surface chemical properties. Physiological factors involved in intestinal absorption of cyclic peptides including CsA has been widely studied, but the data concerning mucins are limited. Previous studies showed that the diffusion coefficients of CsA in mucus using purified porcine gastric or intestinal mucin was lower than an equivalent unstirred aqueous layer^21^. In addition, another study demonstrated that the permeability of CsA across Caco-2 cell monolayers was reduced in the presence of exogenous biosimilar mucus^22^, and it has been hypothesized that mucus layer is a key factor regulating their absorption^5,13^. In terms of molecular size, mucus barriers impede particles larger than their pore size (for gastrointestinal mucus this is estimated as 200 nm)^8^. Therefore, CsA which consists of 11 amino acids (molecular weight 1202.64 Da) is unlikely to be impeded by the molecular sieving properties of the mucus network^23^. However, CsA is a neutral, lipophilic cyclic peptide (Log P = 3.64) and its potential aggregation of mucins likely occurs by interaction with the protein-rich regions of mucins rather than via binding to the negatively charged mucin glycans. Indeed, our data support CsA binding to the N-and C-terminal protein-rich, mucin polymerization domains resulting in non-covalent cross-links that facilitate aggregation of mucin polymers. This interaction alters the mucin network and increases mucus viscosity^19^, which will have a critical regulatory effect on the drug diffusion through the mucus layer. We speculate that the interaction of CsA with mucin is a leading cause of the poor and variable bioavailability reported for CsA (10–60%)^23^.

We have shown that there is a concentration-dependence of CsA–MUC5B interaction that likely induces MUC5B aggregation. Moreover, by varying the concentration of CsA and MUC5B, we have shown that the relative concentration of CsA and MUC5B determine the extent of aggregation. When considering the drug diffusion through a mucus layer, it should be noted that the mucin concentration and mucus porosity is different at the various mucosal surfaces of the body. Even in the gastrointestinal tract, the mucin concentration in mucus varies along its length which would affect mucus viscosity and its binding capacity. Therefore, our results indicate that the interaction of MUC5B with CsA can occur in mucus layers at different mucosal surfaces in the body which have different mucin composition and viscoelastic properties, and this type of interaction may also occur in the intestinal mucus layer. Other mucosal surfaces, which are also routes for drug administration, show distinct expression of MUC2, MUC5AC and MUC5B^11^. Considering these differences and our data, the influence of mucin mediated drug interactions will likely be different at different mucosal surfaces. Therefore, it is important to clarify the molecular mechanisms of mucin-drug interactions, in order to design and develop novel cyclic peptide therapeutics with absorption profiles optimized for specific mucosal surfaces.

We have demonstrated that CsA has ability to induce aggregation of N-terminal and C-terminal regions of MUC5B and that the aggregation of the N-terminal region, but not the C-terminal region, was affected by pH. This differential pH effect could be related to the structures of these domains. The structure of NT5B, which is a dimer, was shown to be an open-boomerang shape mediated centrally by disulfide links between monomers^24^. This open-structure may provide easy access for CsA that then facilitates self-interaction, indeed at low pH in the presence of calcium, N-terminal dimers form higher multimers without CsA^25^. In contrast, the structure of the C-terminal dimer was shown to be a more stable asymmetric, elongated and twisted disulfide-linked structure^26^, suggesting more limited access to CsA and may explain the lack of an effect of pH. The N- and C-terminal domains of MUC5B, which are involved in mucin polymer formation, are highly homologous in sequence and structure to other polymeric mucins such as MUC2 and MUC5AC^27,28^. Therefore, the CsA-induced aggregation of MUC2 and MUC5AC shown here may also be mediated by N-and C-terminal regions of these two mucins. While we do not rule out that mucin O-glycans have a role in the interaction, the effects we report identify protein domain properties and are unlikely to be controlled by the glycosylation, which is mainly in different mucin extended domains. However, we recognize that the pattern of mucin O-glycans is cell line (and tissue specific) and linked to mucin heterogeneity and diversity^7^, therefore their effects on interaction such as that with CsA may be different depending on the source of the mucin. Considering our findings, it is likely that polymeric mucins can interact with other cyclic peptides, but further studies would be required to elucidate such mucin-drug interactions.

To the best of our knowledge, this is the first report of the interaction between purified human gel-forming mucins and a cyclic peptide. It remains an open question of how CsA induces changes in mucin sedimentation properties, and why DAP and POL do not, at least under the conditions studied here. As part of its structure, CsA has partially N-methylated amide groups which may allow intramolecular hydrophobic interactions that could expose hydrogen-bond donors and acceptors to potentially hydrophobic areas of the mucin N- and C-terminal protein domains^29^. N-methylated amide groups are not present in DAP and POL. Davies and coworkers previously proposed that the hydrophobic galloyl groups of green tea polyphenols are essential for interaction with mucin^19^. Other therapeutic agents have also been shown to aggregate gel-forming mucins and potentially limit their bioavailability. For example, vancomycin, a tricyclic glycopeptide antibiotic, interacts with bovine gastric mucins and causes their aggregation, but this interaction is likely driven by electrostatic interactions with negatively charged mucin glycans, such as sialic acid^30^.

In conclusion, we have shown that the interaction of purified MUC5B with cyclic peptide CsA is mediated by N- and C-terminal domains under physiological conditions. However, the precise molecular details remain to be elucidated. Our data provide new insight into the effects of cyclic peptides on the polymeric mucin-created mucus gel network and suggest that mucins are an important physiological factor regulating mucosal permeation of cyclic peptides in vivo. It is by unraveling the effect of cyclic peptide on the supramolecular structure of polymeric mucin that we would gain new insight into what determines importance of mucus physiological properties in the intestinal absorption process of cyclic peptide.

## Methods

### Materials

Daptomycin (DAP), polymyxin B sulfate (POL), and cyclosporin A (CsA) were obtained from Tokyo Chemical Industry Co., Ltd. (Tokyo, Japan). Cesium chloride (CsCl) was purchased from Melford (Ipswich, UK). All other reagents were of analytical grade.

### Cell-line secreted mucin collection

To purify MUC2, human colon adenocarcinoma LS174T cells (European Collection of Cell Culture, Salisbury, U.K), which produce the polymeric mucin MUC2, were grown in Minimum Essential Medium Eagle (MEM) supplemented with 100 units/mL of penicillin/streptomycin, 1% (v/v) of L-glutamine, and 10% fetal bovine serum (FBS), at 37 °C in 5% CO_2_. To purify MUC5AC and MUC5B, knock down A549 cells which were kindly provided by Dr. Mehmet Kesimer (The University of North Carolina)^26,31^, were grown in RPMI-1640 medium, supplemented with 100 units/mL of penicillin/streptomycin, 1 μg/mL puromycin, 1% (v/v) of l-glutamine, and 10% FBS, at 37 °C in 5% CO_2_.

Cells were passaged to 80% confluency in triple layer flasks (Nunc, 500 cm^2^), and then transferred to serum free conditions. Mucin-enriched conditioned media was collected (approximately 4 L for MUC2, and 2 L for MUC5AC and MUC5B) and stored at 4 °C. Conditioned cell media was concentrated five- to tenfold by ultrafiltration using 100 kDa molecular weight cut-off (MWCO) membrane (Vivaflow 200 cassette, Sartorius).

### Purification of polymeric mucins

Two-step isopycnic density gradient centrifugation was used to purify the native polymeric mucins, as previously described^32^. Briefly, mucins were dissolved in CsCl/0.1 M NaCl and subjected twice to density gradient centrifugation (starting density of 1.4 g/mL and then 1.5 g/mL) for 65 h at 40,000 rpm, 15 °C using a Beckman L-90 ultracentrifuge (Beckman Ti45 rotor). Fractions were analyzed for density, absorption at 280 nm, and mucin containing fractions were detected using mucin specific antibodies (MUC2: MAN-2I^33^, MUC5AC: MAN-5ACI^34^, and MUC5B: EUMUC5B^35^) after slot blot onto nitrocellulose.

Mucin-enriched fractions were pooled and dialyzed into 0.1 M NaCl to remove CsCl then purified by size exclusion chromatography on a Sepharose CL2B column (80 cm × 23 mm), eluted with 0.1 M NaCl. In addition, mucin molecular weight distribution was analyzed using multi-angle laser light scattering with size exclusion chromatography (SEC/MALLS) according to well established protocols^34^.

### Production and purification of recombinant MUC5B protein sub-domains

An N-terminal construct (including the D1-D2-D’-D3 domains, residues 26–1304; NT5B, MW of dimer ~ 325 kDa and monomer ~ 175 kDa), C-terminal construct (including the D4-B-C-CK domains, residues 4958–5766; CT5B, MW of dimer ~ 245 kDa and monomer ~ 147 kDa), and the seventh CysD domain (residues 4128–4228; Cys7, MW; ~ 20 kDa) of MUC5B were created and recombinant proteins expressed as previously described^24,26^. Following nickel-affinity purification, the recombinant proteins were size fractionated on a Superose 6 10/300 column (GE Healthcare, UK) (for NT5B and CT5B) and a Superdex 75 10/300 GL column (GE Healthcare, UK) (for Cys7), followed by anion exchange chromatography on a 1 mL Resource Q column (eluted with a gradient of 0–0.5 M NaCl in 25 mM Hepes pH 7.4). The recombinant protein concentrations were determined by bicinchoninic acid (BCA) assay, and molecular weights were measured by SDS-PAGE (Fig. S7).

### Sedimentation analysis of mucins

To assess mucin sedimentation behavior in the presence of cyclic peptides, purified mucins (MUC2, MUC5AC and MUC5B) or MUC5B protein sub-domains were incubated at 37 °C for 1 h in the presence of 1 mM cyclic peptide (dissolved in 10% DMSO). The sedimentation profile of the polymeric mucins and protein sub-domains were determined using rate-zonal centrifugation on sucrose density gradients, as described previously^36^. Sucrose gradient (10–35% (w/v); purified polymeric mucins or 5–20% (w/v); NT5B, CT5B and Cys7) were prepared from charcoal filtered sucrose solutions (dissolved in PBS pH 7.4). 500 μL samples were layered on to the gradients and centrifuged at 210,000*g* (40,000 rpm, 15 °C) for 1 h 15 min (purified mucins) or 2 h 30 min (recombinant protein sub-domains). After centrifugation, sucrose gradients were fractionated into 23 fractions from the top. Any pelleted material was recovered from the bottom of the tube by solubilization with 6 M urea. Fractions were then analyzed for mucin distribution by immunodetection. Although the changes in sedimentation profiles of MUC2 were detectable by using the MUC2 specific antibody MAN-2I, the signal intensities were low (Fig. S8). Therefore, we performed PAS staining^37^ to detect MUC2 in the sucrose gradients samples.

### Mucin detection

#### Slot blot

Samples of equal volume (up to 500 μL) were loaded onto the nitrocellulose membranes using a Minifold II 72 well slot blot apparatus with a water suction vacuum, then analyzed by immunodetection or PAS staining.

#### Immunodetection

The immunodetection was carried out according to previous reports^38^. Briefly, nitrocellulose membranes were blocked in 5% (w/v) milk in 1X Tris buffered saline-Tween (TBST) for 1 h at room temperature. Membranes were then washed in TBST and incubated at room temperature overnight with the MUC2 rabbit polyclonal antiserum, MAN-2I, the MUC5AC rabbit polyclonal antiserum, MAN-5ACI, the MUC5B monoclonal antibody, EUMUC5B, and the mouse anti-6X His-tag monoclonal antibody [Cat# ab15149, RRID:AB_301694; Abcam, MA, USA] (for His-tagged MUC5B protein domains, NT5B and CT5B) all at a dilution of 1:2,000 in 1X TBST. Blots were then washed and incubated with fluorophore-conjugated secondary antibody [IRDye 800 goat anti-mouse (Cat# 926-32210, RRID:AB_621842) or IRDye 800 goat anti-rabbit (Cat# 926-32211, RRID:AB_621843); LI-COR Biosciences, Cambridge, UK] at a dilution of 1:25,000 in TBST for 60 min. Finally, each blot was imaged using a LI- COR Odyssey® CLx Infrared Imaging System.

#### PAS staining

The glycoprotein containing fractions were identified by PAS staining, as previously described^37^. Briefly, nitrocellulose membranes were incubated in a 1% (v/v) periodic acid / 3% (v/v) acetic acid for 30 min. The membrane was then washed in ddH_2_O and incubated for 10 min in a 0.1% (w/v) sodium metabisulfite/0.01 M HCl. Staining was developed using Schiff’s reagent for 10–20 min (until bands appear), and subsequently stopped by washing the membrane for 2 min. The membrane was then rinsed with 0.1% (w/v) sodium metabisulfite / 1 mM HCl and washed in ddH_2_O. Finally, membranes were air dried and were scanned using the BioRad ChemiDoc MP imaging system.

### SDS–PAGE

Electrophoresis was performed on NuPAGE 4–12% (w/v) gels with NuPAGE MOPS SDS running buffer (ThermoFisher Scientific, UK) at 180 V for 40–45 min. Gels were stained with InstantBlue (Expedeon Ltd, UK, or silver stained, and gels were scanned using the BioRad ChemiDoc MP imaging system.

### Measurement of fluorescence spectra

Fluorescence emission spectra of purified MUC5B were measured in the range of 300–450 nm (λ_em_) and the excitation wavelength fixed at 280 nm (λ_ex_) using a Horiba FluoroMax 4 spectrometer (HORIBA scientific, Jobin Yvon, USA). A constant concentration of purified MUC5B (10 μg/mL) was analyzed with increasing concentrations of the CsA (0 to 200 μM). The same concentration range of CsA without MUC5B was used as a control. Samples were left to equilibrate for 30 min at 37 °C and then fluorescence emission spectra were measured.

### Statistical analysis

Data are presented as mean ± standard error of the mean (s.e.m.). Statistical significance between groups was analyzed using Student’s t-test or one-way analysis of variance (ANOVA) followed by Dunnett’s method, and *P* < 0.05 was considered statistically significant. For this statistical analysis was performed with GraphPad Prism v9.2.0.

## Supplementary Information

Supplementary Information.
