# Supplementary material for: The lipophilic cyclic peptide cyclosporin A induces aggregation of gel-forming mucins
Source: Sci Rep. 2022 Apr 13;12:6153. doi: 10.1038/s41598-022-10125-y (PMC9008041; doi:10.1038/s41598-022-10125-y)
Supplement: Supplementary file 1 — Supplementary Information. [file 41598_2022_10125_MOESM1_ESM.docx]

**Supplementary information for**

**The lipophilic cyclic peptide cyclosporin A induces aggregation of gel-forming mucins**

Hisanao Kishimoto^*^, Caroline Ridley, and David J. Thornton^*^

* Corresponding authors:

Hisanao Kishimoto

e-mail: kisimoto@toyaku.ac.jp

David J. Thornton

e-mail: dave.thornton@manchester.ac.uk

**This PDF file includes;**

Total numbers of pages: 11, supplemental figure: 9, table: 5


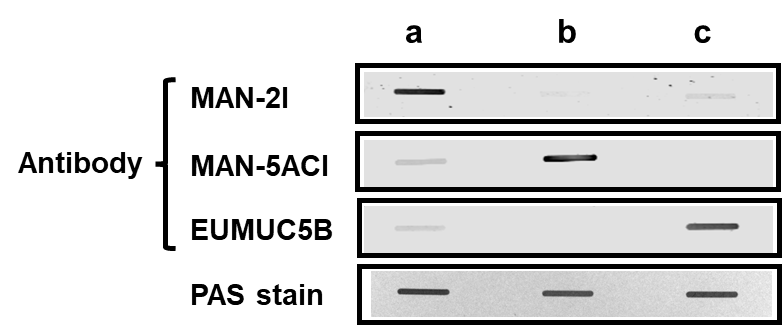


**Figure S1.** Mucins present after purification from LS174T (a), MUC5B-KD A549 (b) and MUC5AC-KD A549 cell lines (c). Mucins were detected by the mucin-specific antibody probes (MUC2: MAN-2I, MUC5AC: MAN-5ACI, and MUC5B: EUMUC5B) or PAS staining. 2 μg of each purified mucin sample was slot blotted onto a nitrocellulose membrane. The blots were cropped and full-length blots are presented in Supplementary Figure S9.


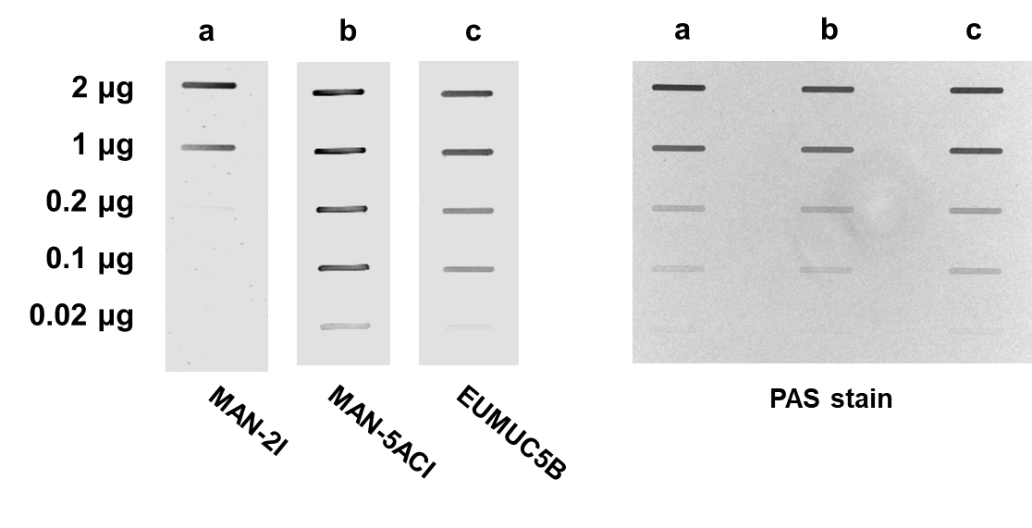


**Figure S2.** The relative detection level of mucins with the specific antibodies. Different amounts of mucins (0.02-2 μg) purified from LS174T (a), MUC5B-KD A549 (b) and MUC5AC-KD A549 cell lines (c) were slot blotted onto a nitrocellulose membrane. Mucins were detected by the mucin-specific antibody probes (MUC2: MAN-2I, MUC5AC: MAN-5ACI, and MUC5B: EUMUC5B) or PAS staining. The blots were cropped and full-length blots are presented in Supplementary Figure S9.


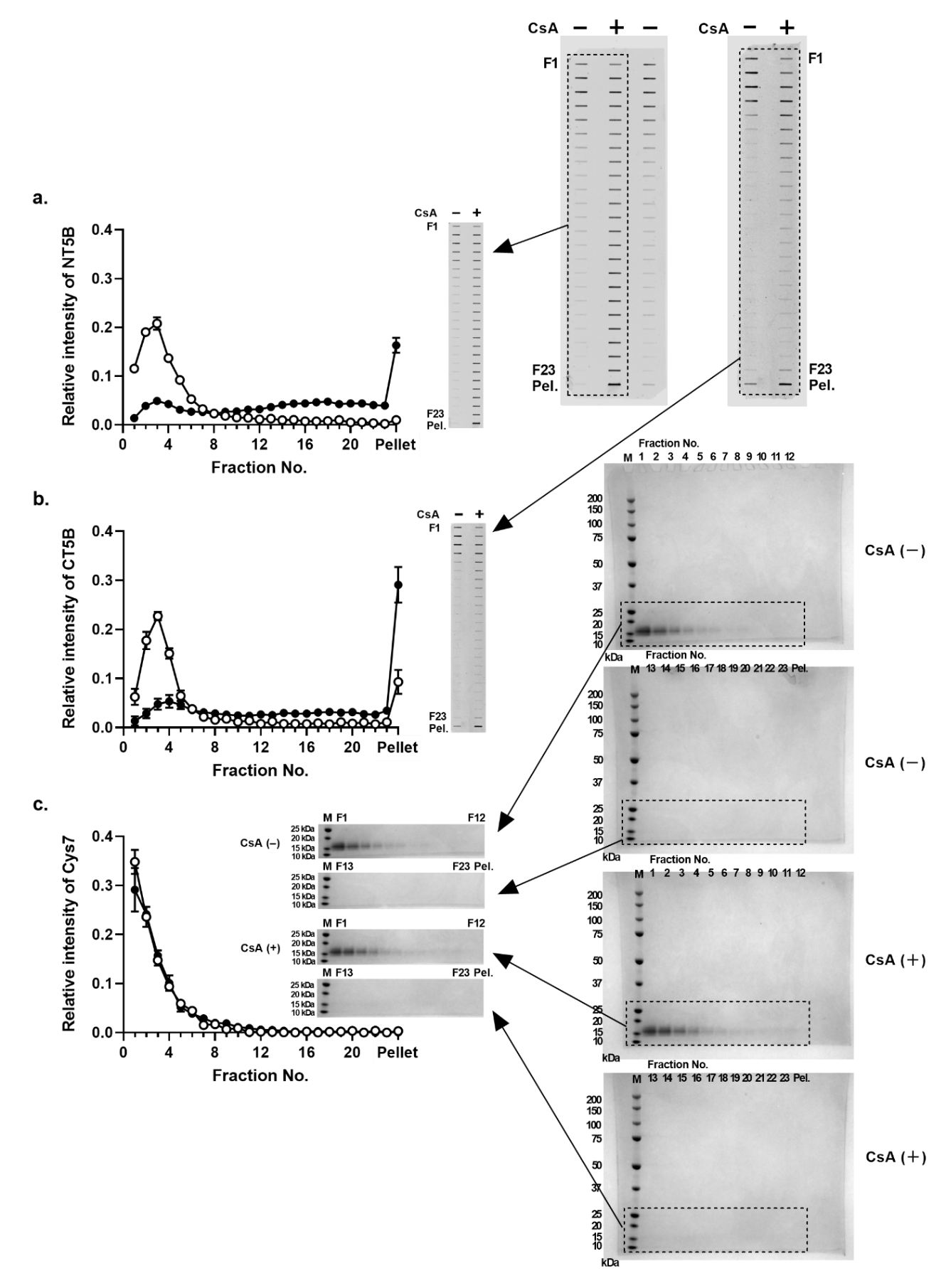


**Figure S3.** Full-length images of representative blots and gels presented in Figure 4. These blots were measured the results from 2 or 3 experiments (2x24 or 3x24 well) using a Minifold II 72 well slot blot apparatus. Each blot and gels were automatically imaged using a LI- COR Odyssey® CLx Infrared Imaging System or a BioRad ChemiDoc MP imaging system, and the protein bands shown in Figure 4 are indicated by dotted boxes.


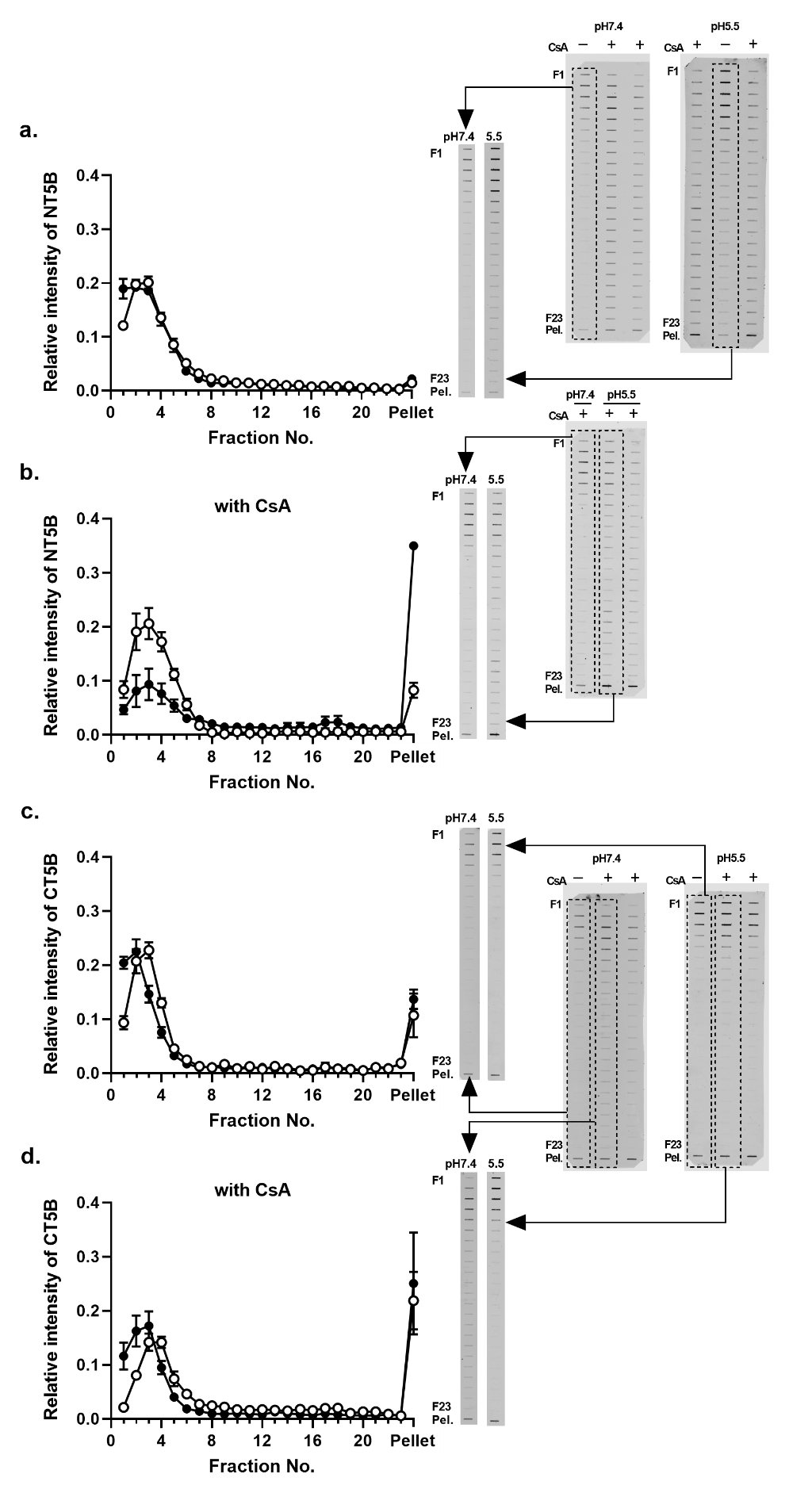


**Figure S4.** Full-length images of representative blots presented in Figure 5. These blots were measured the results from 3 experiments (3x24 well) using a Minifold II 72 well slot blot apparatus. Each blot was automatically imaged using a LI- COR Odyssey® CLx Infrared Imaging System and the protein bands shown in Figure 5 are indicated by dotted boxes.


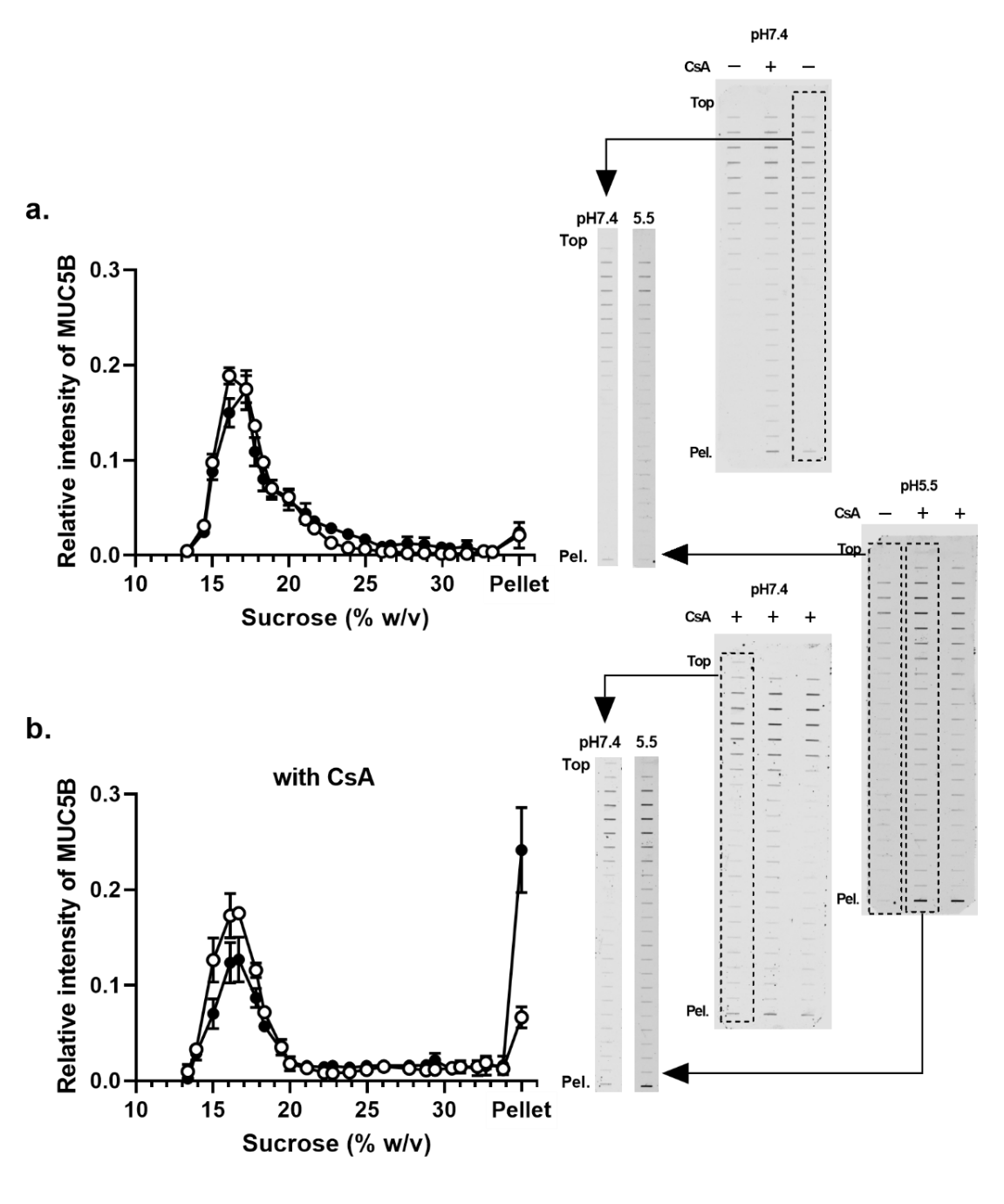


**Figure S5.** Full-length images of representative blots presented in Figure 6. These blots were measured the results from 3 experiments (3x24 well) using a Minifold II 72 well slot blot apparatus. Each blot was automatically imaged using a LI- COR Odyssey® CLx Infrared Imaging System and the protein bands shown in Figure 6 are indicated by dotted boxes.


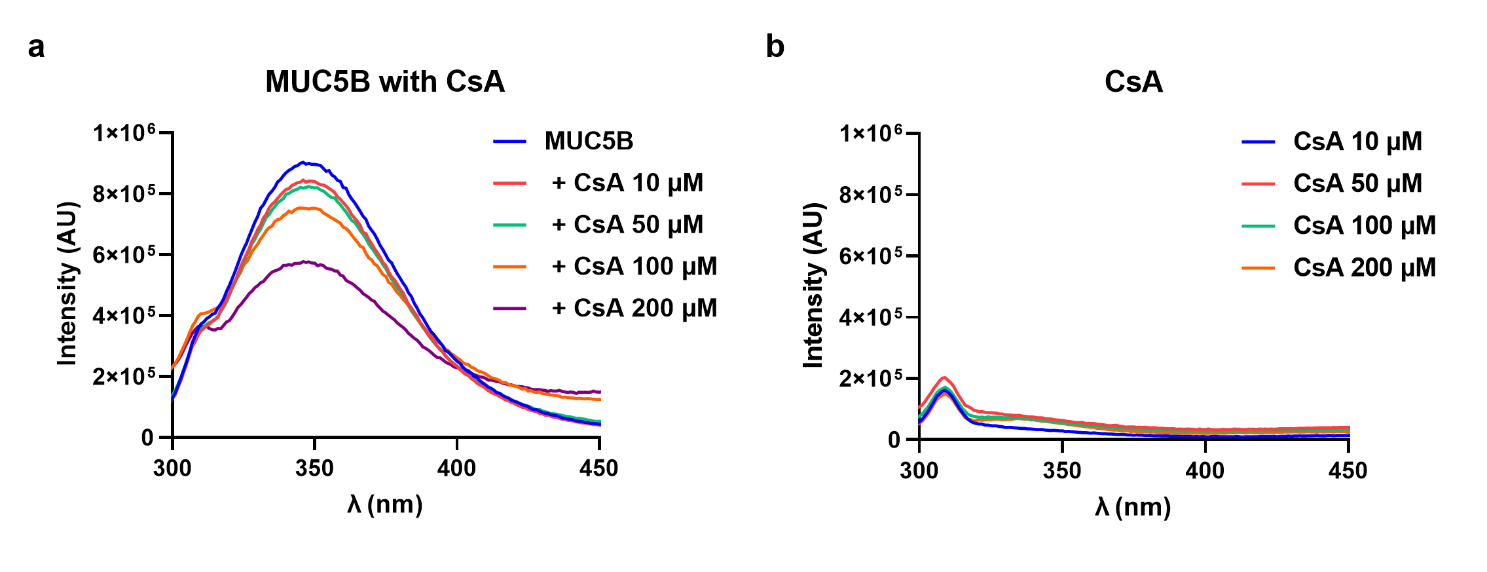


**Figure S6.** (a) Fluorescence emission spectra of purified MUC5B (10 μg/mL) treated with CsA (0, 10, 50, 100 and 200 μM). (b) Fluorescence emission spectra of CsA alone at the concentrations used in (a).


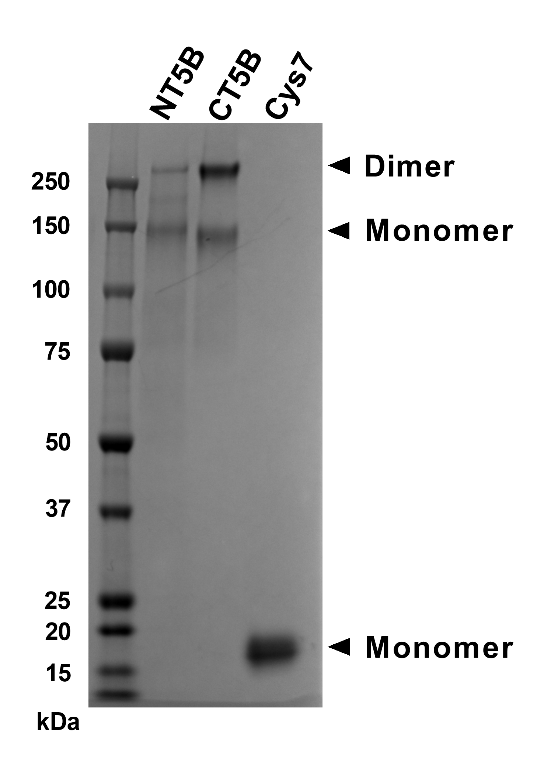


**Figure S7.** Recombinant protein sub-domains of MUC5B were run on SDS-PAGE gel and InstantBlue staining, and gels were scanned using the BioRad ChemiDoc MP imaging system. The gels were cropped and original images of gels are presented in Supplementary Figure S9.


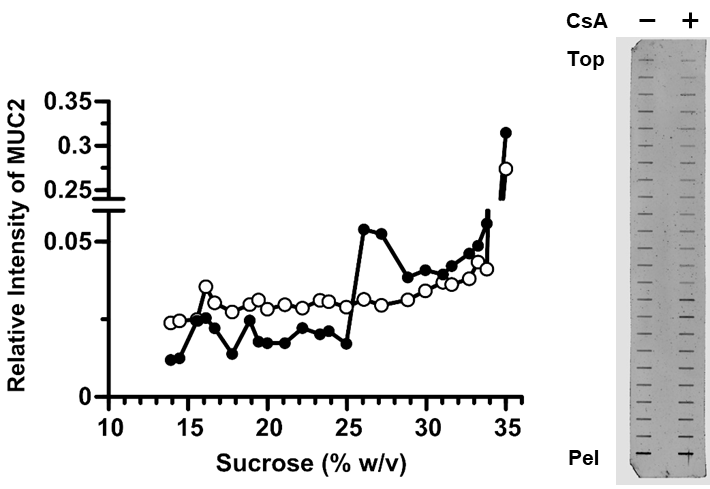


**Figure S8.** Rate-zonal centrifugation in 10–35% (w/v) sucrose gradients of purified MUC2 (200 μg/mL) alone (white circles) or with 1 mM CsA (black circles). Mucins were detected in sucrose gradient fractions after slot blotting using the MUC2-specific antibody probe, MAN-2I. Band intensities were quantified using the Odyssey Imaging system. This blot displayed with high contrast. Due to the low signal obtained, this blot is displayed with high contrast. Top = lowest % sucrose, Pel = pelleted material at the bottom of the tube.


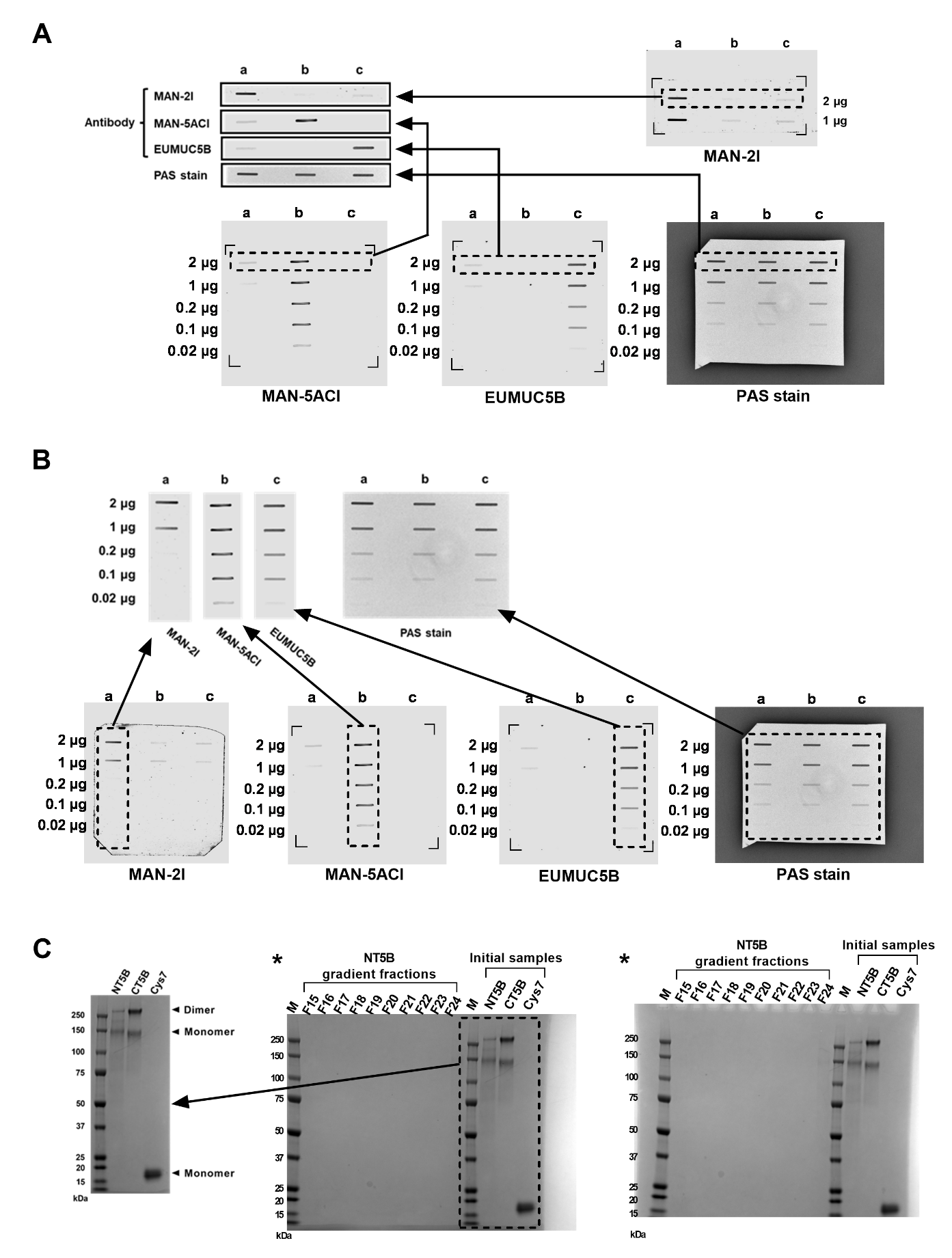


**Figure S9.** Full-length images of blots presented in Figure S1 (A) and S2 (B) and original images of gels presented in Figure S7 (C). Each blot and gels were automatically imaged using a LI- COR Odyssey® CLx Infrared Imaging System or a BioRad ChemiDoc MP imaging system, and the protein bands shown in Figure S1, S2 and S7 are indicated by dotted boxes. *These gel images are scanned at another angle of the same gel and scanned retaining molecular-weight standard bands. M = Molecular-weight standards (kDa).

**Table S1: Distribution of MUC2 across the sucrose gradient with and without cyclic peptides**

| Sucrose  (% w/v) | Percentage of MUC2 in Rate-Zonal Fractions | | | | | | | |
| --- | --- | --- | --- | --- | --- | --- | --- | --- |
|  | Control | | Daptomycin | | Polymyxin B | | Cyclosporin A | |
| 10 ~ 15 | 2.30 | ± 0.78 | 2.78 | ± 0.73 | 0.38 | ± 0.17 | 0.74 | ± 0.50 |
| 15 ~ 20 | 39.91 | ± 0.25 | 44.53 | ± 1.32^*^ | 42.34 | ± 1.14 | 29.01 | ± 0.31^**^ |
| 20 ~ 25 | 23.97 | ± 0.83 | 24.11 | ± 0.58 | 21.26 | ± 1.23 | 22.88 | ± 0.90 |
| 25 ~ 30 | 15.04 | ± 0.23 | 12.79 | ± 0.52 | 15.43 | ± 0.94 | 23.13 | ± 0.36^**^ |
| 30 ~ 35 | 10.98 | ± 0.64 | 8.52 | ± 1.15 | 11.97 | ± 1.03 | 9.63 | ± 0.25 |
| Pellet | 7.80 | ± 0.79 | 7.26 | ± 1.01 | 8.64 | ± 0.37 | 14.61 | ± 0.43^**^ |

Results are presented as the mean ± s.e.m. (n = 3) from 3 independent experiments. **P* < 0.05, ***P* < 0.01 compared with control condition (ANOVA followed by Dunnett’s method). The percentage of MUC2 in fractions across the sucrose gradient was calculated from Figure 1.

**Table S2:** **Distribution of MUC5AC across the sucrose gradient with and without cyclic peptides**

| Sucrose  (% w/v) | Percentage of MUC5AC in Rate-Zonal Fractions | | | | | | | |
| --- | --- | --- | --- | --- | --- | --- | --- | --- |
|  | Control | | Daptomycin | | Polymyxin B | | Cyclosporin A | |
| 10 ~ 15 | 5.48 | ± 0.29 | 3.40 | ± 0.18^**^ | 4.72 | ± 0.29 | 2.60 | ± 0.23^**^ |
| 15 ~ 20 | 59.42 | ± 1.03 | 37.54 | ± 1.24^**^ | 59.82 | ± 1.43 | 30.99 | ± 2.81^**^ |
| 20 ~ 25 | 19.89 | ± 0.76 | 28.86 | ± 0.36^**^ | 22.30 | ± 0.82 | 22.57 | ± 0.28 |
| 25 ~ 30 | 7.36 | ± 0.27 | 17.54 | ± 1.21^**^ | 6.45 | ± 0.24 | 18.33 | ± 0.77^**^ |
| 30 ~ 35 | 4.29 | ± 0.40 | 9.05 | ± 0.96 | 3.27 | ± 0.47 | 17.34 | ± 2.32^**^ |
| Pellet | 3.56 | ± 0.46 | 3.60 | ± 0.47 | 3.43 | ± 0.28 | 8.18 | ± 1.58^*^ |

Results are presented as the mean ± s.e.m. (n = 3–5) from 3 independent experiments. **P* < 0.05, ***P* < 0.01 compared with control condition (ANOVA followed by Dunnett’s method). The percentage of MUC5AC in fractions across the sucrose gradient was calculated from Figure 1.

**Table S3: Distribution of MUC5B across the sucrose gradient with and without cyclic peptides**

| Sucrose  (% w/v) | Percentage of MUC5B in sucrose gradient fractions | | | | | |
| --- | --- | --- | --- | --- | --- | --- |
|  | Control | | Daptomycin | | Polymyxin B | |
| 10 ~ 15 | 10.24 | ± 3.51 | 15.14 | ± 3.17 | 14.34 | ± 2.35 |
| 15 ~ 20 | 74.07 | ± 5.58 | 66.96 | ± 2.6 | 68.13 | ± 4.84 |
| 20 ~ 25 | 10.41 | ± 3.27 | 10.93 | ± 3.25 | 9.68 | ± 3.11 |
| 25 ~ 30 | 2.25 | ± 0.82 | 3.06 | ± 0.67 | 3.40 | ± 1.05 |
| 30 ~ 35 | 1.83 | ± 1.03 | 2.65 | ± 1.19 | 3.07 | ± 1.7 |
| Pellet | 1.20 | ± 0.25 | 1.26 | ± 0.25 | 1.36 | ± 0.18 |

Results are presented as the mean ± s.e.m. (n = 3–5) from 3 independent experiments. **P* < 0.05, ***P* < 0.01 compared with control condition (ANOVA followed by Dunnett’s method). The percentage of MUC5B in fractions across the sucrose gradient was calculated from Figure 1.

**Table S4: Distribution of NT5B and CT5B across the sucrose gradient with 200 μM CsA at pH 5..5 and pH 7.4**

| Fraction  number | NT5B with CsA 200 μM | | | |  | CT5B with CsA 200 μM | | | |
| --- | --- | --- | --- | --- | --- | --- | --- | --- | --- |
|  | pH7.4 | | pH5.5 | |  | pH7.4 | | pH5.5 | |
| 1 ~ 5 | 76.45 | ± 8.47 | 35.09 | ± 9.25 ^*^ |  | 45.88 | ± 3.63 | 58.53 | ± 9.23 |
| 6 ~ 10 | 8.56 | ± 2.22 | 10.77 | ± 0.88 |  | 13.58 | ± 1.57 | 6.15 | ± 0.72 |
| 11 ~ 15 | 2.38 | ± 1.17 | 6.69 | ± 3.46 |  | 8.19 | ± 0.89 | 4.73 | ± 0.81 |
| 16 ~ 20 | 2.52 | ± 2.02 | 8.87 | ± 3.98 |  | 7.78 | ± 0.86 | 3.64 | ± 0.95 |
| 21 ~ 23 | 1.83 | ± 1.55 | 3.62 | ± 1.78 |  | 2.71 | ± 0.28 | 1.91 | ± 0.44 |
| Pellet | 8.26 | ± 1.41 | 34.95 | ± 0.47 ^**^ |  | 21.86 | ± 5.31 | 25.04 | ± 9.43 |

Results are presented as the mean ± s.e.m. (n = 3–5) from 3 independent experiments. **P* < 0.05, **p < 0.01 compared with pH7.4 condition (ANOVA followed by Dunnett’s method). The percentage of NT5B and CT5B in fractions across the sucrose gradient was calculated from Figure 5b and d.

**Table S5: Distribution of MUC5B across the sucrose gradient with and without 200 μM CsA at pH 5..5 and pH 7.4**

| Sucrose  (% w/v) | Control | | | |  | CsA 200 μM | | | |
| --- | --- | --- | --- | --- | --- | --- | --- | --- | --- |
|  | pH7.4 | | pH5.5 | |  | pH7.4 | | pH5.5 | |
| 10 ~ 15 | 3.53 | ± 0.30 | 2.75 | ± 0.47 |  | 4.27 | ± 0.87 | 3.10 | ± 0.67 |
| 15 ~ 20 | 82.56 | ± 0.81 | 72.74 | ± 3.89 |  | 71.51 | ± 3.81 | 51.64 | ± 6.65 |
| 20 ~ 25 | 9.30 | ± 0.81 | 14.64 | ± 2.19 |  | 5.04 | ± 1.04 | 7.47 | ± 1.33 |
| 25 ~ 30 | 1.45 | ± 0.33 | 4.24 | ± 1.82 |  | 6.44 | ± 0.65 | 8.16 | ± 1.95 |
| 30 ~ 35 | 1.05 | ± 0.16 | 3.20 | ± 1.99 |  | 6.11 | ± 1.03 | 5.94 | ± 3.34 |
| Pellet | 2.10 | ± 1.34 | 2.43 | ± 0.57 |  | 6.64 | ± 1.09 | 24.15 | ± 4.45 ^*^ |

Results are presented as the mean ± s.e.m. (n = 3–6) from 3 independent experiments. **P* < 0.05 compared with control condition (ANOVA followed by Dunnett’s method). The percentage of MUC5B in fractions across the sucrose gradient was calculated from Figure 6.
